# A Straining Heart: Transthyretin Amyloidosis as a Cause of Heart Failure

**DOI:** 10.7759/cureus.50957

**Published:** 2023-12-22

**Authors:** Joana Tender-Vieira, Claudemira Pinto, Paula Matias, Pedro Marques, Jorge S Almeida

**Affiliations:** 1 Internal Medicine, Centro Hospitalar Universitário de São João, Porto, PRT; 2 Medicine, Faculdade de Medicina da Universidade do Porto, Porto, PRT

**Keywords:** heart failure, diagnosis, global longitudinal strain, amyloidosis, transthyretin

## Abstract

Cardiac amyloidosis is a disease caused by the deposition of amyloid fibrils in the extracellular space of the heart, most often by immunoglobulin light chains or by transthyretin. It is often underdiagnosed because the signs and symptoms are nonspecific or due to the false perception that the diagnosis always requires an endomyocardial biopsy. Transthyretin amyloidosis is being increasingly recognized as a cause of heart failure, particularly in patients with heart failure with preserved ejection fraction (HFpEF). We present the clinical case of an 86-year-old man whose diagnosis was based on signs and symptoms compatible with cardiac amyloidosis and in which imaging performed a preponderant role. This case reminds clinicians to consider the diagnosis in older patients with HFpEF, left ventricular hypertrophy and rhythm disturbances. It highlights the importance of evaluating global longitudinal strain (GLS) in a standard echocardiographic evaluation.

## Introduction

Amyloidosis is a heterogeneous group of rare disorders characterized by the aberrant aggregation of amyloid proteins in various organs. These proteins are endogenously produced, undergo misfolding (due to genetic mutations, aging or environmental factors) and form insoluble amyloid fibrils. These fibrils accumulate in affected organs, such as the heart, kidneys, liver, nerves, gastrointestinal tract, lungs, muscles, skin and soft tissues [1].

Cardiac amyloidosis is caused by the deposition of amyloid fibrils in the extracellular space of the heart. The amyloid fibrils that most commonly affect the heart are misfolded monoclonal immunoglobulin light chains - AL amyloidosis (AL) or genetic or wild-type transthyretin - transthyretin amyloidosis (ATTR). Other less common amyloid proteins, such as serum amyloid A protein or apolipoprotein A-I, have also been reported in the literature [2,3].

ATTR can be inherited as an autosomal dominant trait caused by pathogenic variants in the transthyretin (TTR) gene or by deposition of wild-type transthyretin protein (ATTRwt), previously called senile cardiac amyloidosis [4]. The initial manifestations of ATTR can be cardiac or extracardiac such as carpal tunnel syndrome, spinal stenosis and biceps tendon rupture. Wild-type TTR amyloid deposits are found in about one-third of older adults undergoing carpal tunnel decompression [5]. Cardiac manifestations include restrictive cardiomyopathy with reduced compliance and impaired diastolic filling, culminating in heart failure. Additionally, amyloid deposits disrupt normal electrical conduction, leading to arrhythmias, especially atrial fibrillation, ventricular arrhythmias and conduction abnormalities [6]. Thickening of ventricular walls, the involvement of heart valves and pericardium and can further exacerbate heart failure in ATTR amyloidosis. The diagnostic approach is conditioned by the clinical presentation and initial imaging findings, particularly on echocardiography. If there exists a discrepancy between the left ventricular thickening (wall thickness ≥ 14 mm) detected in the echocardiogram and the QRS voltage observed in the ECG it should prompt consideration for ATTR [7].

We report the clinical case of an 86-year-old man whose diagnosis of ATTR amyloidosis was established through the observation of signs and symptoms consistent with the condition, with a significant contribution from imaging studies.

## Case presentation

An 86-year-old man with a history of bilateral carpal tunnel syndrome treated surgically 15 years previously, arterial hypertension without known hypertension-mediated organ damage and heart failure with preserved ejection fraction (HFpEF) was admitted to the emergency department due to syncope. The patient also reported complaints of asthenia that had evolved for several days, as well as dyspnea and edema of the lower limbs that had progressively worsened in the previous weeks. He had a clinical history of dysautonomia with a positive orthostatic hypotension test. He had no complaints of chest pain, peripheral neuropathy or other clinical features suggestive of amyloidosis.

**Figure 1:**
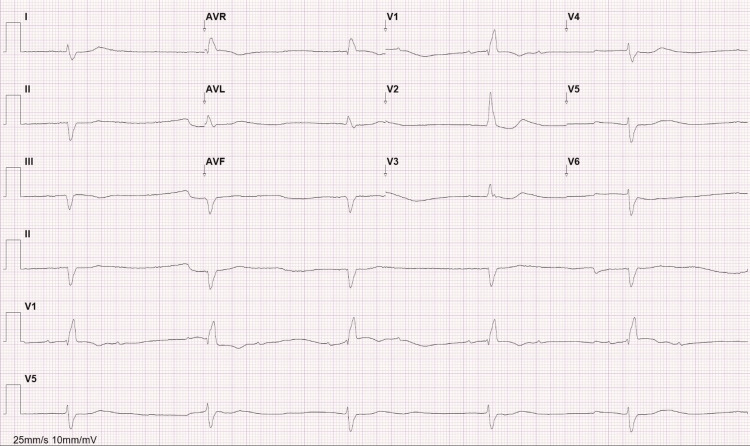
Electrocardiogram performed at admission

The electrocardiogram (ECG) performed showed a 2:1 new atrioventricular block associated with a known right bundle branch block and anterior fascicular block (Figure 1). Due to the new symptomatic disorder in an elderly patient, a permanent pacemaker was placed.

**Figure 2:**
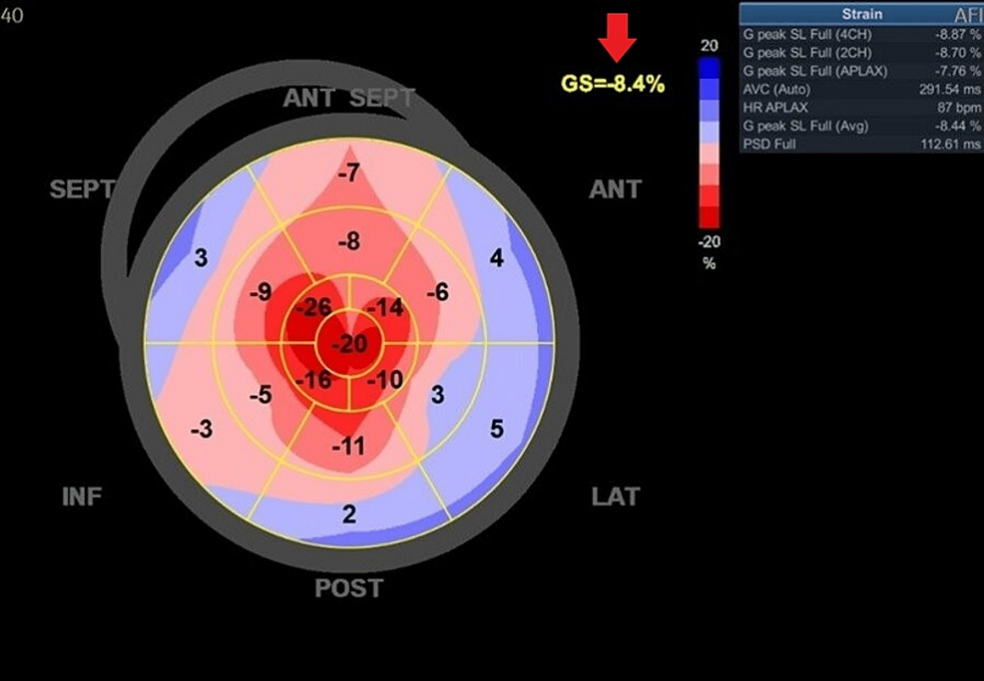
Global longitudinal strain in a patient with TTR amyloidosis showing a characteristic heart-shaped apicobasal gradient. The arrow shows a global longitudinal strain (GLS) markedly reduced (-8.4%).

Laboratory tests did not reveal anemia, sodium, potassium and thyroid function were within reference values. He had a grade 1 acute kidney injury according to the Acute Kidney Injury Network (AKIN) interpreted in the context of type 1 cardiorenal syndrome. B-type natriuretic peptide (BNP) was elevated with a value of 609 pg/mL (reference value <100 pg/mL) and troponin I reached a maximum value of 393 ng/L (reference value < 30 ng/mL). Transthoracic echocardiography (TTE) revealed a concentric left ventricular hypertrophy (left ventricular mass index of 143g/m^2^, a posterior wall thickness of 1.4 cm and a relative wall thickness of 0.67), a small pericardial effusion, no relevant valvular changes and a preserved biventricular function. Global longitudinal strain (GLS) was markedly reduced (-8.4%) and showed the characteristic pattern of apical sparing (Figure 2). These data made diagnoses such as hypertrophic cardiomyopathy or left ventricular hypertrophy associated with hypertension less likely.

**Figure 3:**
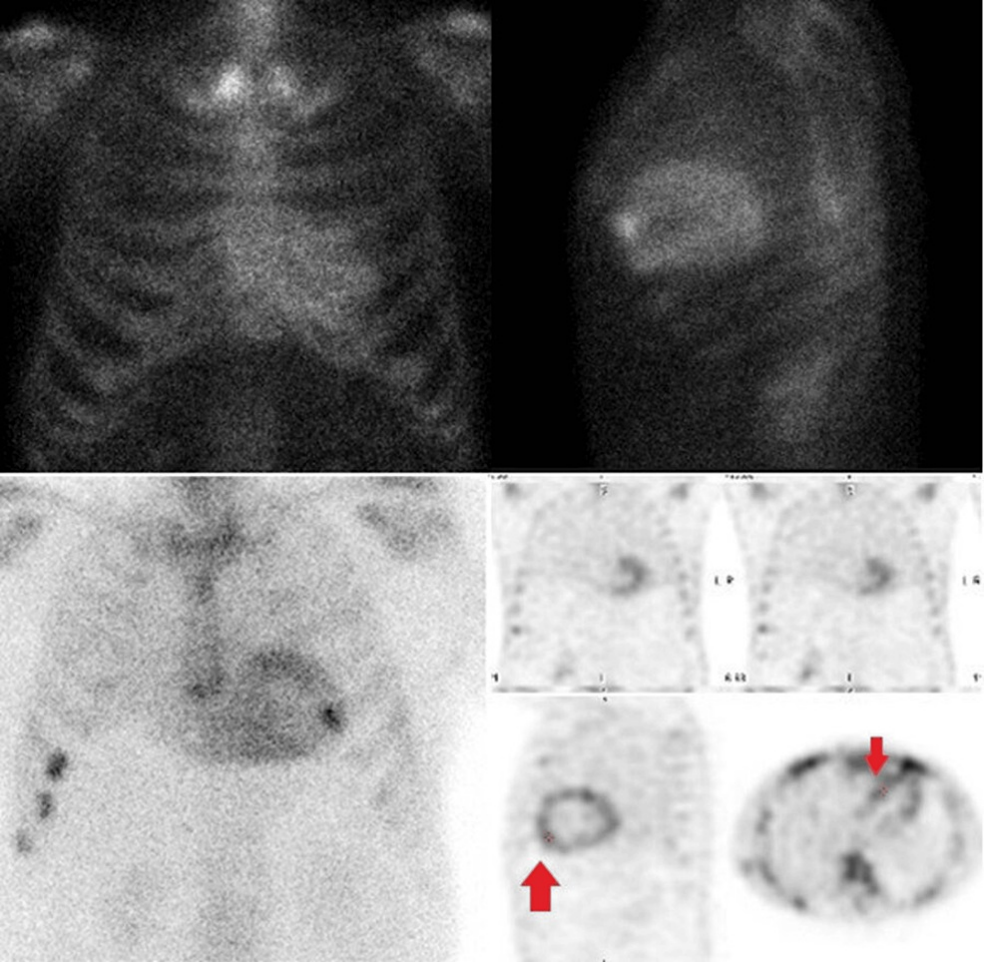
Bone scintigraphy using technetium-99m with increased cardiac uptake (Perugini score of 2) Red arrows show elevated cardiac uptake of technetium-99m.

Etiologic investigation with serum and urine protein electrophoresis and immunofixation excluded light-chain amyloidosis. Bone scintigraphy using technetium-99m exhibited a Perugini score of 2 (Figure 3), consistent with ATTR. Genetic testing was negative for the most common pathogenic variants in the TTR gene.

The patient was discharged medicated with furosemide 120 mg/day and spironolactone 25 mg/day. He was referred to an outpatient clinic for heart failure, where the therapeutic possibility of tafamidis will be evaluated.

## Discussion

The true prevalence of ATTR is difficult to estimate because it is frequently underrecognized due to the attribution of the presenting signs and symptoms to aging, hypertension or HFpEF or even also due to the false perception that the diagnosis can be made only through endomyocardial biopsy [7].

There are some signs and symptoms that are red flags for the diagnosis of amyloidosis. In the case of our patient, in addition to male gender and age, we can refer to carpal tunnel syndrome and dysautonomia with documentation of a positive orthostatic hypotension test and conduction disturbance on the ECG. Natriuretic peptides and troponin T and I levels are commonly increased in these patients, with BNP often disproportionately elevated considering the degree of heart failure. These markers are useful for risk stratification and in assessing treatment response and disease progression [8].

TTE is an important part of the diagnosis of these entities and adds prognostic information regarding the follow-up and management of these patients [9]. GLS is one of the parameters that has been shown to be altered in the earlier stages of cardiac involvement, is associated with greater amyloid burden and has prognostic importance. The frequency of ordering GLS assessments depends on the clinical context, the patient's medical history, and the presence of specific risk factors or symptoms. Inclusion of GLS on echocardiogram may be indicated in situations of high clinical suspicion for conditions like cardiac amyloidosis, hypertrophic cardiomyopathy or connective tissue diseases and for monitoring disease progression in certain cases. Cardiac magnetic resonance imaging (CMR) is more sensitive and specific than TTE, allowing the diagnosis of cardiac amyloidosis even before the development of left ventricular hypertrophy [10]. In patients with suspected amyloidosis by these imaging methods, it is recommended to perform serum and urine protein electrophoresis and immunofixation in order to exclude AL amyloidosis. Bone scan with technetium-99m allows us to obtain the Perugini score based on the degree of radiopharmaceutical uptake. The uptake of this product is zero or very low in cases of AL amyloidosis [6,7]. Endomyocardial biopsy is not always necessary for the diagnosis of cardiac amyloidosis. As seen in our patient, it is possible to establish the diagnosis non-invasively as long as we have a cardiac image (TTE or CMR) with findings compatible with amyloidosis, grade 2 or 3 on the Perugini score and absence of monoclonal protein deposition. Abdominal fat aspiration allows a histological diagnosis to be made, revealing the green birefringence pattern under polarized light of Congo red‐stained amyloid deposits, which is pathognomonic of amyloidosis. However, the sensitivity of this diagnostic method in the case of ATTR is much lower compared to AaL [6,9].

Despite our patient's age, genetic testing for the transthyretin gene was requested. This should be performed in all patients because of the implications for other family members and for helping to predict the impact in other organs, since there are mutations associated with specific clinical manifestations [8,9].

ATTR treatment is based on measures to control heart failure symptoms with loop diuretics and mineralocorticoid receptor antagonists, conduction disorders with pacemaker placement or rhythm disturbances with an implantable cardioverter-defibrillator, and also on prognosis-modifying therapies. There are three groups of these modifying therapies: stabilizing drugs that prevent TTR misfolding, such as tafamidis, which can be used in New York Heart Association (NYHA) functional class I to III patients; silencing drugs that affect protein synthesis, such as patisiran and inotersen, used in patients with hereditary ATTR, and degraders that remove amyloid deposits, which are under investigation. Liver transplantation may be indicated in patients with hereditary ATTR although in some cases the heart disease still progresses [6,11,12].

There are several studies that associate certain baseline risk factors with the progression of the disease, but they do not yet include new therapies. These patients should be evaluated in an outpatient clinic with ECG, troponin and BNP every six months, and TTE and 24h Holter-ECG annually [6].

## Conclusions

The management of ATTR involves a comprehensive approach targeting heart failure symptoms and conduction disorders, and utilizing prognosis-modifying therapies such as tafamidis, patisiran and inotersen. Regular outpatient evaluations, incorporating various diagnostic measures, are essential for monitoring disease progression and adjusting treatment strategies in this field.

## References

[1] Muchtar E, Dispenzieri A, Magen H (2021). Systemic amyloidosis from A (AA) to T (ATTR): a review. J Intern Med.

[2] Benson MD, Buxbaum JN, Eisenberg DS (2018). Amyloid nomenclature 2018: recommendations by the International Society of Amyloidosis (ISA) nomenclature committee. Amyloid.

[3] Rauf MU, Hawkins PN, Cappelli F (2023). Tc-99m labelled bone scintigraphy in suspected cardiac amyloidosis. Eur Heart J.

[4] Gertz MA, Benson MD, Dyck PJ (2015). Diagnosis, prognosis, and therapy of transthyretin amyloidosis. J Am Coll Cardiol.

[5] Sekijima Y, Uchiyama S, Tojo K (2011). High prevalence of wild-type transthyretin deposition in patients with idiopathic carpal tunnel syndrome: a common cause of carpal tunnel syndrome in the elderly. Hum Pathol.

[6] Garcia-Pavia P, Rapezzi C, Adler Y (2021). Diagnosis and treatment of cardiac amyloidosis: a position statement of the ESC Working Group on Myocardial and Pericardial Diseases. Eur Heart J.

[7] Kittleson MM, Maurer MS, Ambardekar AV (2020). Cardiac amyloidosis: Evolving diagnosis and management: a scientific statement from the American Heart Association. Circulation.

[8] Castiglione V, Franzini M, Aimo A (2021). Use of biomarkers to diagnose and manage cardiac amyloidosis. Eur J Heart Fail.

[9] Gillmore JD, Maurer MS, Falk RH (2016). Nonbiopsy diagnosis of cardiac transthyretin amyloidosis. Circulation.

[10] Knight DS, Zumbo G, Barcella W (2019). Cardiac structural and functional consequences of amyloid deposition by cardiac magnetic resonance and echocardiography and their prognostic roles. JACC Cardiovasc Imaging.

[11] Patel RK, Fontana M, Ruberg FL (2021). Cardiac amyloidosis: multimodal imaging of disease activity and response to treatment. Circ Cardiovasc Imaging.

[12] Maurer MS, Schwartz JH, Gundapaneni B (2018). Tafamidis treatment for patients with transthyretin amyloid cardiomyopathy. N Engl J Med.

